# Determinants of Birth Intervals Using Prentice-Williams-Peterson-Gap Time Model: Tehran Case Study

**DOI:** 10.22074/IJFS.2021.134701

**Published:** 2021-06-22

**Authors:** Arezoo Bagheri, Mahsa Saadati

**Affiliations:** National Institute for Population Research, Tehran, Iran

**Keywords:** Birth Interval, Fertility, Survival Analysis

## Abstract

**Background:**

Total fertility rate (TFR) in Iran decreased from the year 2000 and recently Iran has experienced fertility rates below replacement level. Birth interval is one of the most important determinants of fertility levels and plays a vital role in population growth rate. Due to the importance of this subject, the aim of this study was analyzing three birth intervals using three Survival Recurrent Event (SRE) models.

**Materials and Methods:**

In a 2017 cross-sectional fertility survey in Tehran, 610 married women, age 15-49 years, were selected by multi-stage stratified random sampling and interviewed using a structured questionnaire. The effects of selected covariates on first, second and third birth intervals were fitted to the data using the Prentice-WilliamsPeterson-Gap Time (PWP-GT) SRE model in *SAS 9.4*.

**Results:**

Calendar-period had a significant effect on all three birth intervals (P<0.01). The Hazard Rate (HR) for a short birth interval for women in the most recent calendar-period (2007-2017) was lower than for the other calendarperiods. Women’s migration influenced second (P=0.044) and third birth intervals (P=0.031). The HR for both birth intervals in migrant women was 1.298 and 1.404 times shorter, respectively than non-migrant women. Women’s employment (P=0.008) and place of residence (P<0.05) also had significant effects on second birth interval; employed women and those living in developed, completely-developed and semi-developed areas, compared to unemployed women and those living in developing regions, had longer second birth intervals. Older age at marriage age increased the HR for a short third birth interval (P<0.01).

**Conclusion:**

The analysis of birth interval patterns using an appropriate statistical method provides important information for health policymakers. Based on the results of this study, younger women delayed their childbearing more than older women. Migrant women, unemployed women and women who live in developing regions gave birth to their second child sooner than non-migrant employed women, and women who lived in more developed regions. The implementation of policies which change the economic and social conditions of families could prevent increasing birth intervals and influence the fertility rate.

## Introduction

Fertility influences population size and distribution, so analyses of fertility behavior provide important information for policy makers who plan population control and evaluate family planning programs (1). Family planning programs in Iran in the past two decades were aimed at fertility reduction and had reduced the total fertility rate (TFR) to 2.01 by 2016 (2, 3). In recent years, government and policy makers have applied new pronatalist policies to increase fertility. The success of such policies rely on understanding the determinants of low fertility.

Among the different indicators used to identify fertility patterns, such as number of children borne to each woman, birth interval is very important. The pattern of birth intervals not only denotes the pace of child bearing but also increases the chances of transition to higher parity (4). Many studies have shown that long birth intervals lead to a low fertility rate and decreased population growth (5). Since birth interval plays an important role in the health of mothers and children, it also merits special attention in public health. Birth interval has become one of the main strategies in health promotion programs for mothers and children in the last 20 years in Iran (6). Consequently, in recent years, many studies have examined the interval between marriage and first birth, and interbirth intervals. Most of the research has focused on first birth interval (FBI) because of its advantages; women do not forget details of their first pregnancy, and the delay in the menstrual cycle that occurs after subsequent fertilizations is not observed. Furthermore, if FBI is short (<12 months) and occurs at a young age, subsequent pregnancies may happen faster and the fertility rate will be increased (7). Reduction of child mortality (8), increasing levels of education for women and their children (7), and balancing individual and family goals (9) are influential factors that affect first childbearing. Saadati et al. (10- 12) showed that in Tehran and Semnan, calendar-period, place of residence, social insecurity, educational level, and employment had significant effects on women’s FBI.

In addition to delayed childbearing, long inter-birth intervals (>75 months) can lead to a below-replacement level TFR (13-15). Many studies have considered determinants of long birth intervals; Soltanian et al. (16) showed that there were significant effects on birth intervals by women’s age at first marriage, parental education, women’s employment, use of contraceptives, and number of live births. Erfani et al. (5, 13-15) showed that several factors, such as woman’s calendar-period, marriage age, contraceptive method, educational level, employment, place of residence and household income influenced women’s first, second and third birth intervals in Tehran and Hamedan.

Due to its simplicity, the proportional hazards Cox model is used to analyze birth intervals in many studies in Iran and around the world (5, 6, 13, 14, 17, 18). Cox models can determine the relationship between the HR and covariates without specifying the baseline hazard function. The assumption underlying the validity of the Cox model is the proportionality of the hazards, or independence of event times, a fact often ignored in applications of this model. However, in most studies, including those on birth intervals, event times (births) are correlated. In these studies, using Cox models which ignore the correlations between birth intervals may lead to errors in estimating the standard deviation of the desired parameters and result in incorrect inferences (19). In such cases, SRE models, which allow for the given event (e.g. birth) to occur more than once for each individual and that include the correlations between events to be included in the model, should be used (19, 20). SRE models include Anderson-Gill (AG), Wei-Lin-Weissfeld (WLW), PWP-Total Time (TT), PWP-GT, and frailty models which should be selected for use based on the research objective, and the nature of the data (19).

According to the last census (2016), Tehran, Gilan and Mazandaran had the lowest TFRs in Iran; 1.38, 1.51, and 1.56, respectively (21), underlining the importance of studying the fertility behavior of women who live in Tehran. As birth interval is such an important determinant of women’s fertility, the aim of the present study was to determine socio-demographic factors that affected women’s first, second and third birth intervals in 2017 (22). In order to attain valid results the PWP-GT model was used to analyze the data. Data collection and statistical methods are described next, findings from the models fitted are illustrated in results, and some concluding remarks are given in the discussion and conclusion sections.

## Materials and Methods

This study used data from a 2017cross-sectional survey “Effects of socio-economic rationality dimensions on childbearing behavior in Tehran” (22). All married women aged 15-49 years were eligible. The final sample included 610 women from Tehran province selected using multi-stage sampling (23). The structured questionnaire collected demographic data, fertility history and attitudinal factors related to childbearing. Based on the aims of this study, only demographic and fertility history questions were considered. 10 demographers and sociologists confirmed the validity of questionnaire, and its reliability was verified by a Cronbach’s Alpha of at least 0.771.

Participants provided oral consent to participate in this study and the Ethical code was supplied by National Population Studies and Comprehensive Management Institute for the questionnaire (20/18627) (22). Birth intervals, defined as the length of time between two successive live births, were considered the response outcome of interest. Since very few women had more than 3 children, only three birth intervals, marriage to first, first to second, and second to third births were included in this survey. Data for nulliparous women and women with one or two children were considered as censored for the first, second, and third birth intervals, respectively (Table 1).

According to different studies devoted to the investigation of influential factors for birth intervals in Iran, the most important socio-demographic covariates, also analyzed in this study, are age at first marriage (5, 14, 24- 26), educational level (9, 10, 25, 27, 28), couple’s educational level (26, 28), employment (5, 26, 28), region of residence (14, 25), Internal migration (5, 14, 15), family expenditure (13, 15, 26), and calendar-period (5, 13-15, 29). Four calendar-periods were used in the present study, before May 1987, May 1987 - April 1997, May 1997 - April 2007, and May 2007 - April 2017, to cover the years during which the study participants would have given birth. These ten-year periods are assumed to measure to some extent the socio-economic changes and major policies that have taken place during these periods (13, 14).

To evaluate the influence of selected covariates on birth intervals accurately, PWP-GT SRE models were used to analyze the data in SAS 9.4.

### Statistical methods

Recurrent event data refer to sequential events that occur more than once. As mentioned before, childbearing is an example of recurrent event data. Many studies have analyzed birth intervals based on conventional models which may provide misleading results. Conventional analysis of the FBI using a Cox model is described in Equation (1):

h_i_(t)=h_0_(t) exp (βX_i_),i=1,…,n

Where h_i_ (t) denotes the hazard given the covariate values for the i^th^ subject and survival time (t). The term h_0_ (t) is called the baseline hazard; it is the hazard for the respective individual when the values of all the covariates are equal to zero. **β** is the vector of regression coefficients, and x_i_ is the vector of covariates for the i^th^ subject.

**Table 1:** Frequency distribution and median birth intervals in months (in parentheses) of women by selected covariates


Covariate		1^st^ Birth	2^nd^ Birth	3^rd^ Birth

Calendar- period				
	Before May 1987	5.1 (31)	7.0 (28)	10.8^a^
	May 1987-Appril 1997	17.5 (38)	28.3 (57)	32.4^a^
	May 1997-Appril 2007	33.7 (35)	42.6 (65)	36.5^a^
	May 2007-Appril 2017	43.7 (40)	22.1 (41)	20.3^a^
Marriage age (Y)				
	<16	9 (31)	14.8 (40)	25.7 (70)
	17-19	19.9 (37)	27.7 (61)	33.8 (45)
	20-24	41.8 (40)	36.3 (57)	27.0 (65)
	25-29	22.5 (39)	16.4 (51)	10.8 (51)
	30+	6.9 (38)	4.7 (43)	2.7 (31)
Educational level				
	Primary and less	6.5 (31)	10.8 (36)	23.6 (70)
	Secondary and high school	9.1 (31)	14.0 (41)	19.4 (57)
	Diploma	45.0 (37)	50 (60)	48.6 (55)
	B.Sc./Associate	30.2 (42)	20.4 (61)	6.9 (66)
	M.Sc. and Ph.D.	9.1 (38)	4.8 (48)	1.4^a^
Couple’s educational level				
	Primary and less	6.3 (33)	9.6 (41)	20.8 (70)
	Secondary and high School	14.8 (31)	20.0 (40)	26.3 (47)
	Diploma	36.2 (37)	38.8 (58)	34.7 (58)
	B.Sc./Associate	29.8 (42)	22.0 (64)	15.2 (60)
	M.Sc. and Ph.D.	12.9 (37)	9.6 (48)	2.8 (30)
Woman’s employment				
	Unemployed	28.6 (37)	20.2 (63)	8.1^a^
	Employed	71.4 (42)	79.8 (52)	91.9^a^
Migration				
	Non-migrant	86.9 (38)	89.7^a^	93.0^a^
	Migrant	13.1 (40)	10.3^a^	7.0^a^
Family expenditure (each month)				
	Less than 2 million Tomans	56.6 (37)	63.5^a^	72.9^a^
	2-3.5 million Tomans	32.2 (41)	27.3^a^	24.3^a^
	More than 3.5 million Tomans	11.2 (38)	9.2 (48)	2.9^a^
Region of residence				
	Developing	16 (41)	12.4 (63)	4.1 (31)
	Semi-developed	15.4 (46)	10.5 (43)	6.8 (95)
	Developed	44.1 (37)	46.9^a^	48.6 (60)
	Completely-developed	24.5 (38)	30.2^a^	40.5 (55)
Total exposed to the birth interval (median birth interval)		610 (38)	469 (55)	258 (58)
Total experienced the birth (%)		469 (76.9)	258 (55.0)	74 (28.7)
Total censored (%)		141 (23.1)	211 (44.9)	184 (71.3)


^a^ ; Medians were not computed, as the cumulative survival distribution did not go below 50% or less, which means more than half of women were pregnant but had not yet given birth.

However, in this situation, the results of Cox model are misleading because the model does not take into account all the available data, and the correlation between recurrent event times. Ignoring this correlation leads to misleading results; in this case, confidence interval estimation could be artificially long, as a result the statistical power decreases. Consequently a statistical model that considers the correlations between the data must be applied in these situations (19).

Original Cox models have been extended to deal with recurrent event data. Examples include AG, PWP-TT, PWP-GT, WLW and frailty models (30).

The AG model assumes that the occurrence of the current event is not affected by the previous events, so each subject is at risk of all events over the entire follow-up period. Thus, the baseline hazard is common for all events. In this model risk intervals are considered as (t_0_ , t_1_ ], (t_1_ , t_2_ ] … (t_m_, last follow-up time] for each subject and each recurrent event for the i^th^ subject is assumed to follow Equation (1). This a suitable model when correlations among events for each individual are induced by the measured covariates. Thus, dependence is captured by appropriate specification of the time-dependent covariates, such as number of previous events or some function thereof.

In the WLW model, time intervals are given as (0, t_1_ ], (0, t _2_ ] … (0, last follow-up time] for each subject, and is suitable for studies in which each subject is followed from study entry. In this model, all individuals are at risk of recurrence during the follow up, regardless of the occurrence of previous events, but different baseline hazards for each event are assumed in the model. The hazard function for the k^th^ event of the i^th^ subject is explained by Equation (2):

h_ik_(t)=h_ok_(t)exp(β_k_X_ik_), i=1,...,n ,k=1,...,l

Where, “k” is the number of strata for each person at time t, X_ik_ denotes the predictor variable for i^th^ individual at time t, and β_k_ is the regression coefficient for k^th^ event (strata).

The PWP model analyses recurrent events by stratification, based on the prior number of events during the study. All subjects are at risk for the first event (stratum), but only those who experienced the previous event are at risk for the next event. PWP-TT models have the same outcome as the AG model and evaluate the effect of a covariate for the kth event since entry into the study. In PWP-GT models the outcome is defined as gap time, which is the time since the previous event. So, time intervals are given as (0, t1], (0, t2-t1] … (0, last follow-up time-previous time] for each subject. PWP-GT evaluates the effect of a covariate for the kth event since the time from the previous event.

In PWP-GT models, the hazard function for i^th^ subject, and k^th^ event is described in Equation (3):

h_ik_ (t)=h_0k_ (t-t_k-1_) exp(β_k_ X_ik_),i=1,…,n ,k=1, …, l (3) t-1 denotes the former occurrence time of the event.

Unlike the AG model, the effect of covariates may vary from event to event in the PWP models. If it is reasonable to assume that the occurrence of the first event increases the likelihood of a recurrent event, then PWP would be the recommended model. PWP models (TT or GT) are also indicated when there is interest in estimating effects for each event separately. The PWP models assume that a subject can only be at risk for a given event after he/she has experienced the previous event.

When subject-specific random effects can explain the unmeasured heterogeneity in a model, a frailty model can be applied which leads to a person-specific interpretation of the parameter estimates. In this model production of consistent estimations depends on the number of events, number of subjects and the distribution of events/subject. The Frailty model is described in Equation (4):

h_ik_ (t)=h_0k_ (t) ω_i_ exp(β_k_ X_ik_), i=1, …, n , k=1, …,l (4)

Where, Frailty ω_i_ is the unobserved (random) factors for i^th ^subject.

Selection of the recurrent event models depends on many factors, including number of the events, relationships between subsequent events, effects varying or not across recurrences, biological process, and dependence structure. In this study only women who have already had one or two children can give birth to second and third children; so AG and WLW models are unsuitable for these data. Frailty models were not selected in this study because frailty variances were very low for second and third birth intervals (0.043 and 0.02, respectively). The PWP-GT model was selected instead of the PWP-TT model, because the distribution of children per women is small, and prediction of time to next birth was an outcome of interest (31). 

### Results

Mean age of the women in this study was 35.38±7.91years, and age of first marriage was 22.59 + 4.39 years. Most of women and their husbands had an academic level education (44.3%, 46.4, respectively), “less than 2 million Tomans” family expenditure (56.6%), were unemployed (68%), and lived in developed regions (44.1%). Only 15.7% of women had migrated in last 10 years. Among 610 married women, 21.2%, 34.7%, 31.3%, and 12.8% respectively had 0, 1, 2, and 3 children. Table 1 shows that half of the women had their first birth almost 3 years (38 months) after marriage but spaced their second birth by more than 4 years (55 months). 

Median interval to first birth by educational level showed, as expected, that university-educated women had the longest interval to first birth. In employed women, immigrant women and women who had a family expenditure of 2 to 3.5 million Tomans childbearing was more delayed than among unemployed women, non-migrant women and women who lived in households with other expenditure profiles. 

**Fig.1:**
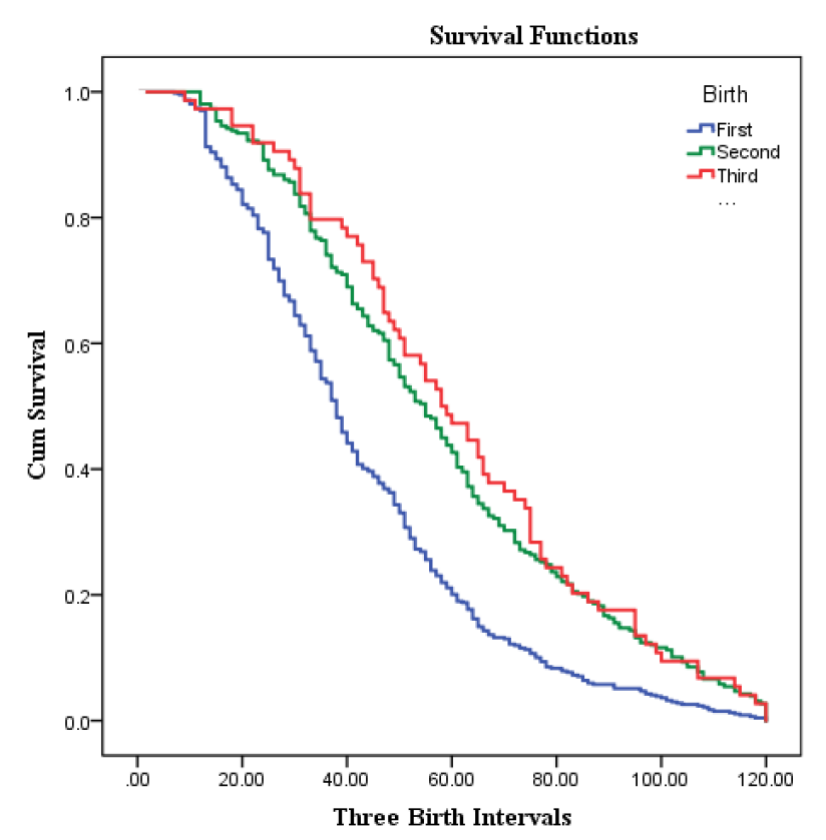
Survival curves for first, second, and third birth intervals

Survival curves based on Kaplan-Meir estimations for women’s first, second, and third birth intervals are shown in Figure 1. As this figure displays, women gave birth to their first child sooner than the second and third one.

Table 2 shows the results of the PWP-GT model for first, second, and third birth intervals based on selected covariates.

The results of the PWP-GT model revealed that calendar-period had a significant effect on all three birth intervals (P<0.01). The largest gap from marriage to first, first to second, and second to third child was among women in the last calendar period. HRs for a short birth interval for first, second, and third children for women in last calendar-period were 0.479, 0.286, and 0.161 times lower than women in first calendar-period. In other words, the HR for short birth intervals decreased from the first to the last calendar-period. Women’s employment and region of residence also affected the second birth interval. Employed mothers were at lower risk of a short interval between first and second child compared to unemployed women (HR=0.758, P=0.008). In other words, the likelihood hazard of having a second child for employed women was less than unemployed women. Women who lived in developed (HR=0.576, P<0.001), completely-developed (HR=0.705, P=0.015), and semi-developed (HR=0.819, P=0.041) regions were less likely to have a short second birth interval than women who lived in developing regions. So, women who lived in developing regions had a greater likelihood of having a second child than women who lived in other regions. HR of women’s deduction in second and third birth intervals for migrant women was 1.298, and 1.404 times than non-migrant women, respectively. Therefore, the likelihood of having a second and third child was greater in migrant women than non-migrant women. Increasing age at marriage was associated with a higher HR for a shorter interval between the second and third birth (HR=1.047, P<0.001).

Recurrent event data structure and how to organize the data for each recurrent event model, and the SAS code for fitting these models are given in the Tables S1, 2 (See Supplementary Online Information at www.celljournal. org), respectively.

**Table 2:** Estimated hazard rate from PWP-GT model assessing the impact of selected covariates on first, second and third birth intervals


Covariate		1^s‌t^ Birth			2^nd^ Birth			3^rd^ Birth		
		HR	SE	P value	HR	SE	P value	HR	SE	P value

Calendar-period	Before May 1987 (ref)									
	May 1987-Appril 1997	0.614	0.266	0.066	0.356	0.212	<.001	0.478	0.235	<.001
	May 1997-Appril 2007	0.755	0.268	0.296	0.308	0.210	<.001	0.190	0.239	<.001
	May 2007-Appril 2017	0.479	0.271	0.006	0.286	0.221	<.001	0.161	0.246	<.001
Marriage age (Y)		0.998	0.012	0.898	1.012	0.010	0.239	1.047	0.012	<.001
Educational level	Primary and less (ref)									
	Secondary and high school	1.287	0.267	0.344	1.078	0.193	0.697	1.215	0.195	0.318
	Diploma	0.812	0.255	0.415	0.855	0.179	0.382	0.981	0.194	0.923
	BS/Associate	0.684	0.277	0.17	0.872	0.207	0.509	1.025	0.243	0.919
	MS and PhD	0.615	0.344	0.158	0.762	0.274	0.322	1.424	0.331	0.285
Couple’s educational level	Primary and less (ref)									
	Secondary and high school	1.214	0.249	0.436	1.074	0.182	0.693	1.109	0.188	0.581
	Diploma	1.017	0.255	0.949	0.896	0.183	0.549	0.924	0.202	0.696
	BS/Associate	0.816	0.269	0.449	0.907	0.195	0.615	0.841	0.225	0.441
Woman’s employment	MS and PhD	1.012	0.319	0.97	0.955	0.243	0.848	0.882	0.288	0.662
	Unemployed (ref)									
Migration	Employed	0.969	0.128	0.804	0.758	0.104	0.008	0.879	0.131	0.325
	Non-migrant (ref)									
Family expenditure (each months)	Migrant	1.062	0.17	0.722	1.298	0.129	0.044	1.404	0.157	0.031
	Less than 2 million Tomans (ref)									
	2- 3.5 million Tomans	1.108	0.127	0.42	1.013	0.096	0.895	1.119	0.113	0.319
Regions of residence	More than 3.5 million Tomans	1.208	0.194	0.329	1.067	0.157	0.680	1.086	0.210	0.693
	Developing (ref)									
	Semi-developed	0.777	0.165	0.125	0.819	0.098	0.041	0.883	0.106	0.242
	Developed	0.65	0.225	0.056	0.576	0.146	0.000	0.768	0.185	0.152
	Completely-developed	0.922	0.199	0.684	0.705	0.143	0.015	0.734	0.189	0.102


ref; Reference group.

## Discussion

According to various studies, birth interval is one of the factors affecting the number of children borne by a woman, with short birth intervals tending to lead to more children (2-5). For this reason the study of birth intervals has become important in Iran.

In most studies in which birth intervals have been analysed, each interval was modelled separately using Cox or parametric survival models regardless of the correlation between them. Rasekh and Momtaz (32) analyzed birth intervals using Cox models without considering correlation between the intervals. Soltani et al. (18) used Cox and Weibull parametric models to examine socio-economic factors affecting first and second birth intervals based on Demographic and Health Study (2000) data in Iran. Cox models assume that intervals are independent, when in fact a woman’s birth intervals are correlated. Ignoring the interdependence of birth intervals cause a bias in estimating the variance of the model’s parameters meaning results for the effects of covariates on the birth intervals are not valid.

In this article, the effect of selected covariates on first, second, and third birth intervals were determined using a PWP-GT SRE model. Based on the fitted model, calendar-period had significant effects on all three birth intervals. Women in the last calendar-period were least likely to give birth to children after a shorter interval than women in the other calendar-periods. While half of the women who were exposed to their first pregnancy before May 1987 gave birth to their first child 37 months after marriage, half of the women who were in last calendar-period (May 2007 to April 2017), delayed childbearing by up to 40 months. The HR for a short interval between ‘first and second’, and ‘second and third’ children decreased in recent calendar-periods. This finding is similar to the results obtained by Erfani et al. (5, 14).

Marriage to FBI has increased over the last three decades. Increasing age at first marriage is associated with an increased HR for a shorter interval between the second and third child. This means that with increasing age at marriage the interval between the birth of the second and third child decreased. This may be due to the shorter remaining fertile period and trying to reach the desired number of children. Many other studies have reported that birth interval decreases as marriage age increases (6, 33, 34). 

The birth interval between first and second child for unemployed women was shorter than for employed women, as in other studies (6, 15, 16). Due to the time required to adapt to their new situation, migrant women are expected to have longer inter-birth intervals compared with non-migrants (15). In this study first birth intervals for migrant women were longer than non-migrant women. On the other hand, migrant women gave birth to their second and third child sooner than non-migrant women.

Region of residence had a significant effect on second birth interval. Women who lived in semi- developed, developed, and completely-developed regions gave birth to their second child later than those living in developing regions. Erfani (13) showed that women who lived in completely- developed regions in Tehran have their second child later than ones who lived in developing regions.

The main advantage of this study is the analysis of birth intervals using the PWP-GT model. In most studies these data are analyzed using Cox or parametric survival models which may lead to incorrect results. This study also has some limitations. Some fertility history factors such as contraceptive use, breast-feeding duration for previous birth, and survival status of previous children were unavailable. These questions will consider in the next survey which will be implemented in the near future.

## Conclusion

Women in the 2007-2017 calendar-period delayed childbearing due to economic and social conditions in society and the current uncertainty. This finding also applied to second and third children. The longer interval between the first and second births of employed women indicates that they have a second child later than unemployed women, and as a result, may experience a lower fertility level. Policymakers can enable women to have children at shorter birth intervals by providing appropriate socio-economic conditions.
